# Assessment of Costs and Care Quality Associated With Major Surgical Procedures After Implementation of Maryland’s Capitated Budget Model

**DOI:** 10.1001/jamanetworkopen.2021.26619

**Published:** 2021-09-24

**Authors:** Oluseyi Aliu, Andrew W. P. Lee, Jonathan E. Efron, Robert S. D. Higgins, Charles E. Butler, Anaeze C. Offodile

**Affiliations:** 1 Department of Plastic Surgery, Johns Hopkins University, Baltimore, Maryland; 2 University of Texas Southwestern Medical School, Dallas; 3 Department of Surgery, Johns Hopkins University, Baltimore, Maryland; 4 Department of Plastic Surgery, University of Texas MD Anderson Cancer Center, Houston; 5 Department of Health Services Research, University of Texas MD Anderson Cancer Center, Houston; 6 Baker Institute for Public Policy, Rice University, Houston, Texas

## Abstract

**Importance:**

In 2014, Maryland implemented the all-payer model, a distinct hospital funding policy that applied caps on annual hospital expenditures and mandated reductions in avoidable complications. Expansion of this model to other states is currently being considered; therefore, it is important to evaluate whether Maryland’s all-payer model is achieving the desired goals among surgical patients, who are an at-risk population for most potentially preventable complications.

**Objective:**

To examine the association between the implementation of Maryland’s all-payer model and the incidence of avoidable complications and resource use among adult surgical patients.

**Design, Setting, and Participants:**

This comparative effectiveness study used hospital discharge records from the Healthcare Cost and Utilization Project state inpatient databases to conduct a difference-in-differences analysis comparing the incidence of avoidable complications and the intensity of health resource use before and after implementation of the all-payer model in Maryland. The analytical sample included 2 983 411 adult patients who received coronary artery bypass grafting (CABG), carotid endarterectomy (CEA), spinal fusion, hip or knee arthroplasty, hysterectomy, or cesarean delivery between January 1, 2008, and December 31, 2016, at acute care hospitals in Maryland (intervention state) and New York, New Jersey, and Rhode Island (control states). Data analysis was conducted from July 2019 to July 2021.

**Exposures:**

All-payer model.

**Main Outcomes and Measures:**

Complications (infectious, cardiovascular, respiratory, kidney, coagulation, and wound) and health resource use (ie, hospital charges).

**Results:**

Of 2 983 411 total patients in the analytical sample, 525 262 patients were from Maryland and 2 458 149 were from control states. Across Maryland and the control states, there were statistically significant but not clinically relevant differences in the preintervention period with regard to patient age (mean [SD], 49.7 [19.0] years vs 48.9 [19.3] years, respectively; *P* < .001), sex (22.7% male vs 21.4% male; *P* < .001), and race (0.3% vs 0.4% American Indian, 2.8% vs 4.5% Asian or Pacific Islander, 25.9% vs 12.7% Black, 4.7% vs 11.9% Hispanic, and 63.5% vs 63.4% White; *P* < .001). After implementation of the all-payer model in Maryland, significantly lower rates of avoidable complications were found among patients who underwent CABG (−11.3%; 95% CI, −13.8% to −8.7%; *P* < .001), CEA (−1.6%; 95% CI, −2.9% to −0.3%; *P* = .02), hip arthroplasty (−0.8%; 95% CI, −1.0% to −0.5%; *P* < .001), knee arthroplasty (−0.4%; 95% CI, −0.7% to −0.1%; *P* = .01), and cesarean delivery (−1.0%; 95% CI, −1.3% to −0.7%; *P* < .001). In addition, there were significantly lower increases in index hospital costs in Maryland among patients who underwent CABG (−$6236; 95% CI, −$7320 to −$5151; *P* < .001), CEA (−$730; 95% CI, −$1367 to −$94; *P* = .03), spinal fusion (−$3253; 95% CI, −$3879 to −$2627; *P* < .001), hip arthroplasty (−$328; 95% CI, −$634 to −$21; *P* = .04), knee arthroplasty (−$415; 95% CI, −$643 to −$187; *P* < .001), cesarean delivery (−$300; 95% CI, −$380 to −$220; *P* < .001), and hysterectomy (−$745; 95% CI, −$974 to −$517; *P* < .001). Significant changes in patient mix consistent with a younger population (eg, a shift toward private/commercial insurance for orthopedic procedures, such as spinal fusion [4.3%; 95% CI, 3.4%-5.2%; *P* < .001] and knee arthroplasty [1.6%; 95% CI, 1.0%-2.3%; *P* < .001]) and a lower comorbidity burden across surgical procedures (eg, CABG: −0.7% [95% CI, −0.1% to −0.5%; *P* < .001]; hip arthroplasty: −3.0% [95% CI, −3.6% to −2.3%; *P* < .001]) were also observed.

**Conclusions and Relevance:**

In this study, patients who underwent common surgical procedures had significantly fewer avoidable complications and lower hospital costs, as measured against the rate of increase throughout the study, after implementation of the all-payer model in Maryland. These findings may be associated with changes in the patient mix.

## Key Points

**Question:**

Did the rates of avoidable complications and hospital costs among surgical patients change after the implementation of Maryland’s all-payer model, which caps hospital expenditures and mandates reductions in avoidable complications?

**Findings:**

In this comparative effectiveness study of 525 262 Maryland patients who received surgery before and after implementation of Maryland’s all-payer model, lower complication rates were observed for coronary artery bypass grafting (11% decrease), carotid endarterectomy (2% decrease), hip arthroplasty (1% decrease), knee arthroplasty (<1% decrease), and cesarean delivery (1% decrease). Smaller estimated changes in hospital costs vs other states were also observed, with increases reduced by $6236 for coronary artery bypass grafting, $730 for carotid endarterectomy, $328 for hip arthroplasty, $415 for knee arthroplasty, $300 for cesarean delivery, and $745 for hysterectomy.

**Meaning:**

This study found that after implementation of the all-payer model in Maryland, the rates of avoidable complications decreased, and a slower increase in hospital costs occurred across an array of surgical procedures.

## Teaser

This comparative effectiveness study uses state inpatient data from the Healthcare Cost and Utilization Project to examine the association between the implementation of Maryland’s all-payer model and the incidence of avoidable complications and resource use among adult surgical patients.

## Introduction

Although health care expenditures in the US constitute nearly one-fifth of economic productivity, the US still lags behind other developed economies in several population health measures.^1,2,3^ As a consequence, policy makers have been supplanting traditional fee-for-service payments with alternative payment models.^4,5,6^

Under a waiver from the Centers for Medicare & Medicaid Services Innovation Center, the state of Maryland implemented the all-payer model in January 2014.^7,8^ This statewide alternative payment model involved total capitation (global budgets) for hospital outlays associated with emergency department, inpatient, and outpatient care. Encompassing all payers (ie, government and private/commercial), the policy required the state to meet spending targets, such as $330 million or more in Medicare savings over 5 years, and quality targets, such as reductions in hospital readmissions and participation in the Maryland Hospital-Acquired Conditions Program (MHACP). Regarding the latter, hospitals were mandated to achieve a 30% cumulative reduction in hospital-acquired conditions (HACs) selected from a list of potentially preventable complications.^8^ Potentially preventable complications were defined as “harmful events…or negative outcomes…that may result from the processes of care and treatment rather than from natural progression of the underlying illness.”^9^^(p65)^

According to a recent evaluation of Center for Medicare & Medicaid Innovation payment models, the all-payer model generated the most financial savings,^10^ and expansion of this model to other states is currently being considered.^11,12^ It is important to evaluate whether the all-payer model achieves the desired goals among surgical patients, who are an at-risk population for most potentially preventable complications.^13^ Furthermore, surgical care episodes constitute a large share of health care expenditures (estimated to be 7.3% of the US gross domestic product in 2025) and are anchored by a hospitalization, which is the focus of the all-payer model.^14^ The all-payer model incentivizes Maryland hospitals to (1) reduce costs (ie, resource use) associated with inpatient surgical admissions and (2) avoid additional costs associated with potentially preventable complications.^15^

Our aim was to examine the association between implementation of Maryland’s all-payer model and changes, if any, in the costs and complication rates of the index hospitalization. We focused on a broad population of surgical patients and hypothesized that implementation of the all-payer model would be associated with significant reductions in both avoidable complications and the rate of increase in index hospitalization costs.

## Methods

### Study Design

A programmatic overview of Maryland’s all-payer model has been published elsewhere.^8,16,17^ This comparative effectiveness study used a difference-in-differences design to compare outcomes and hospitalization costs before (2008-2013) and after (2014-2016) implementation of the all-payer model in Maryland. The study was approved by the Johns Hopkins Hospital Institutional Review Board and deemed exempt from informed consent because publicly available, deidentified data were used. This study followed the International Society for Pharmacoeconomics and Outcomes Research (ISPOR) reporting guideline for comparative effectiveness research.

### Data Source

We used data from the Healthcare Cost and Utilization Project state inpatient databases. Given the statewide implementation of Maryland’s all-payer model, our comparison groups were obtained from states outside of Maryland (intervention state), which included New York, New Jersey, and Rhode Island (control states). In addition, because Maryland adopted Medicaid expansion as part of the Affordable Care Act (at approximately the same time the all-payer model was initiated), we chose these states for comparison because they had similar expansion records and timing (ie, January 1, 2014), which could mitigate any unmeasured confounding from concurrent Medicaid expansion policies. Given the nature of the all-payer model and our focus on the activities associated with the index hospitalization (ie, costs and adverse events), the state inpatient databases captured the most representative and appropriate study population relative to more segmented data sets from federal (eg, Medicare) or private/commercial (eg, Optum or Truven Health MarketScan) payers.

### Study Population

The study sample comprised adult patients (aged ≥18 years) who underwent any of the following elective surgical procedures in an acute care hospital between January 1, 2008, and December 31, 2016: coronary artery bypass grafting (CABG), carotid endarterectomy (CEA), spinal fusion, hip arthroplasty, knee arthroplasty, cesarean delivery, or hysterectomy. These surgical procedures are among the most commonly performed in the US^18,19^ and have been the focus of alternative payment model testing (eg, bundled payments).^20,21^ Patients were identified using the appropriate codes from the *International Classification of Diseases, Ninth Revision, Clinical Modification* or *Procedure Coding System* version (*ICD-9-CM* or *ICD-9-PCS*) and the *International Classification of Diseases, Tenth Revision, Clinical Modification* or *Procedure Coding System* version (*ICD-10-CM* or *ICD-10-PCS*). We excluded emergency procedures given their increased costs and higher risk of major complications.^22^

### Outcomes

Primary outcomes associated with the index hospitalization were costs and incidence of HACs. For hospitalization costs, we used data on total charges for each discharge record obtained from state inpatient databases. The total charge variable from the state inpatient databases represents the amount billed for hospital services; however, this amount may not reflect actual costs. The Healthcare Cost and Utilization Project supplemental files containing hospital-specific cost-to-charge ratios enable conversion of total charges recorded in the state inpatient databases to actual hospital costs, and previous studies of surgical costs have used this measure.^23,24,25,26^

Hospital-acquired conditions were treated as a binary outcome if recorded as present for an observation that had 1 or more of 14 selected avoidable complications identified using *ICD-9* and *ICD-10* diagnostic codes (eTable 1 in the Supplement). We used the diagnosis present on admission indicator to accurately identify patients who developed HACs of interest during admission rather than those who presented with HACs as secondary diagnoses.

We enforced a 6-month waiting period after the date of all-payer policy implementation in Maryland (January 1 to June 20, 2014). Our rationale was that any meaningful change to clinical practice in response to the all-payer model would require time to fully develop, and this waiting period would ensure that our estimates were not associated with the early postpolicy period, a time during which changes in clinical practice had the lowest plausibility of being associated with the policy. This approach was also consistent with previous studies that examined state-level policy interventions (eg, the all-payer model or Medicaid expansion) via a difference-in-differences framework.^27,28^

### Statistical Analysis

Demographic and clinical characteristics of the study population in Maryland and the control states were summarized and compared using a χ^2^ test for categorical variables and a *t* test for continuous variables. Differences in rates of HACs were summarized for the periods before and after implementation of the all-payer model in both Maryland and the control states.

We performed formal testing of the parallel baseline trends assumption in quarterly rates of HACs and mean changes in the costs and complication rates of the index hospitalization for each surgical procedure of interest, comparing Maryland with the control states. To accomplish this analysis, we estimated models (a logistic model for HACs and a generalized linear model with a log link for index hospitalization costs) for the baseline period (ie, before implementation of the all-payer model) including 2 interaction terms, the first between a Maryland/control states indicator and a preintervention time variable (ie, quarterly) and the second between a Maryland/control states indicator and a linear time trend encompassing the preintervention study period.^29^ Of note, the MHACP was originally implemented in 2011, but its impact before implementation of the all-payer model was mediated by its revenue-neutral payment adjustment approach. In addition, all Maryland hospitals were exempt from the Centers for Medicare & Medicaid Services Hospital-Acquired Conditions Reduction Program (HACRP). Hence, for the HAC outcome, we assumed that the initial implementation period of the MHACP in July 2011 may have altered the measured outcomes of the modified MHACP (which was changed to accommodate the all-payer model) in 2014; hence, we used 2 models, pre-2011 and pre-2014, to estimate baseline trends in HACs.

For our 7 surgical procedure categories, we performed a difference-in-differences analysis to determine the change, if any, in each outcome from before and after implementation of the all-payer model across Maryland and the control states. For HACs, the difference-in-differences analyses used multivariate logistic regression models that included a 2-way categorical interaction term for periods before and after implementation of the all-payer model for Maryland and the control states; this interaction term served as the primary parameter of interest.^17^ Patient-level covariates in the adjusted models were identified a priori and included age, sex, race (American Indian, Asian or Pacific Islander, Black, Hispanic, White, or other [the term *other* in the state inpatient databases for New Jersey and Rhode Island refers to individuals of multiracial background such as White and Black, White and Asian, Black and Alaskan Native; New York State did not specify the meaning of *other* before 2014]), primary payer (private/commercial insurance, Medicare, Medicaid, or other), and Elixhauser comorbidity index (0, 1-2, or >2). In a separate conservative model, we included a 2-way categorical interaction term for the pre-2011 MHACP and post-2011 MHACP periods for Maryland and the control states to adjust for consequences of the MHACP before it was redesigned to accommodate the all-payer model. To estimate baseline trends, we included an interaction term between the Maryland/control states indicator and a linear time trend encompassing the entire study period.^17^ Index hospitalization costs were evaluated in a similar manner using generalized linear models with a log link. However, the linear models did not include the interaction term for the pre-2011 and post-2011 MHACP periods because the program that was designed and implemented in 2011 did not include cost as an outcome. Results of models are presented as differences in the change in rates or means with corresponding 95% CIs.

We examined variation in the use of surgical procedures across different patient subgroups, including subgroups by race, primary payer status, and comorbidity burden, that may have been incentivized by all-payer model implementation. We estimated logistic difference-in-differences models using patient characteristics as binary categorical outcome variables, and we used a 2-way categorical interaction term for the periods before and after implementation of the all-payer model for Maryland and the control states as the indicator for each surgical procedure. Results of models were reported as the differences in the change in rates for each characteristic. A 2-tailed *P* < .05 was considered statistically significant, and no adjustments were made for multiple comparisons. All analyses were conducted using Stata software, version 15.1 (StataCorp LLC). Data analysis was conducted from July 2019 to July 2021.

## Results

Of 2 983 411 total patients, 525 262 patients were from Maryland and 2 458 149 were from control states. Across Maryland and the control states, there were statistically significant but not clinically relevant differences in the preintervention period with regard to patient age (mean [SD], 49.7 [19.0] years vs 48.9 [19.3] years, respectively; *P* < .001), sex (22.7% male vs 21.4% male; *P* < .001), and race (0.3% vs 0.4% American Indian, 2.8% vs 4.5% Asian or Pacific Islander, 25.9% vs 12.7% Black, 4.7% vs 11.9% Hispanic, and 63.5% vs 63.4% White; *P* < .001). Additional patient demographic and clinical characteristics before and after implementation of the all-payer model categorized by surgical procedure are shown in Table 1.

**Table 1.** Population Demographic and Clinical Characteristics in Maryland and Control States Before and After Implementation of All-Payer Model by Procedure

Characteristic	Before implementation			After implementation		
	No. (%)		*P* value	No. (%)		*P* value
	Maryland	Control states		Maryland	Control states	
**CABG**						
Total patients, No.	15 804	83 388	NA	6188	34 227	NA
Age, mean (SD), y	65.2 (10.6)	65.9 (10.7)	<.001	66.5 (10.4)	66.4 (10.3)	<.001
Sex						
Male	11 311 (71.6)	61 466 (73.7)	<.001	4607 (74.5)	25 815 (75.4)	.10
Female	4493 (28.4)	21 922 (26.3)		1581 (25.5)	8412 (24.6)	
Race and ethnicity						
American Indian	62 (0.4)	263 (0.3)	<.001	28 (0.5)	106 (0.3)	<.001
Asian or Pacific Islander	341 (2.2)	2994 (3.6)		224 (3.6)	1596 (4.7)	
Black	2560 (16.2)	5361 (6.4)		1090 (17.6)	2240 (6.5)	
Hispanic	257 (1.6)	6359 (7.6)		153 (2.5)	2891 (8.4)	
White	11 768 (74.5)	60 478 (72.5)		4495 (72.6)	23 628 (69.0)	
Other^a^	816 (5.2)	7933 (9.5)		198 (3.2)	3766 (11.0)	
Elixhauser comorbidity index						
0	59 (0.4)	1654 (2.0)	<.001	23 (0.4)	424 (1.2)	<.001
1-2	1690 (10.7)	24 520 (29.4)		671 (10.8)	6576 (19.2)	
>2	14 055 (88.9)	57 214 (68.6)		5494 (88.8)	27 227 (79.5)	
Payer						
Private/commercial insurance	6023 (38.1)	28 578 (34.3)	.09	2610 (42.2)	10 587 (30.9)	.15
Medicare	8041 (50.9)	43 125 (51.7)		3223 (52.1)	18 477 (54.0)	
Medicaid	951 (6.0)	7456 (8.9)		613 (9.9)	4075 (11.9)	
Other	789 (5.0)	4229 (5.1)		192 (3.1)	1088 (3.2)	
**CEA**						
Total patients, No.	10 377	43 938	NA	3604	16 808	NA
Age, mean (SD), y	80.0 (9.7)	71.8 (9.4)	<.001	70.8 (9.9)	71.5 (10.0)	<.001
Sex						
Male	5769 (55.6)	25 895 (58.9)	<.001	2053 (57.0)	9897 (58.9)	.03
Female	4608 (44.4)	18 043 (41.1)		1551 (43.0)	6911 (41.1)	
Race and ethnicity						
American Indian	13 (0.1)	110 (0.3)	<.001	NA^b^	29 (0.2)	<.001
Asian or Pacific Islander	91 (0.9)	649 (1.5)			352 (2.1)	
Black	1156 (11.1)	1763 (4.0)			904 (5.4)	
Hispanic	66 (0.6)	2130 (4.8)			854 (5.1)	
White	8927 (86.0)	37 484 (85.3)			13 694 (5.8)	
Other^a^	124 (1.2)	1802 (4.1)			975 (5.8)	
Elixhauser comorbidity index						
0	300 (2.9)	2431 (5.5)	<.001	105 (2.9)	784 (4.7)	<.001
1-2	3812 (36.7)	22 236 (50.6)		1273 (35.3)	6680 (39.7)	
>2	6265 (60.4)	19 271 (43.9)		2226 (61.8)	9344 (55.6)	
Payer						
Private/commercial insurance	2350 (22.6)	9944 (22.6)	.09	733 (20.3)	3301 (19.6)	.15
Medicare	7522 (72.5)	31 589 (71.9)		2617 (72.6)	12 157 (72.3)	
Medicaid	293 (2.8)	1428 (3.3)		200 (5.5)	1024 (6.1)	
Other	212 (2.0)	977 (2.2)		54 (1.5)	326 (1.9)	
**Spinal fusion**						
Total patients, No.	50 563	178 563	NA	21 275	89 872	NA
Age, mean (SD), y	56.1 (13.7)	54.3 (14.0)	<.001	58.5 (13.4)	56.3 (13.6)	<.001
Sex						
Male	22 818 (45.1)	86 492 (48.4)	<.001	9748 (45.8)	44 120 (49.1)	<.001
Female	27 745 (54.9)	92 071 (51.6)		11 527 (54.2)	45 752 (50.9)	
Race and ethnicity						
American Indian	77 (0.2)	422 (0.2)	<.001	64 (0.3)	189 (0.2)	<.001
Asian or Pacific Islander	501 (1.0)	2994 (1.7)		282 (1.3)	1649 (1.8)	
Black	9394 (18.6)	17 838 (10.0)		4784 (22.5)	10 031 (11.2)	
Hispanic	747 (1.5)	13 571 (7.6)		449 (2.1)	7019 (7.8)	
White	38 864 (76.9)	131 664 (73.7)		15 366 (72.2)	64 065 (71.3)	
Other^a^	980 (1.9)	12 124 (6.8)		330 (1.6)	6919 (7.7)	
Elixhauser comorbidity index						
0	8202 (16.2)	51 218 (28.7)	<.001	2949 (13.9)	20 989 (23.4)	<.001
1-2	23 368 (46.2)	86 729 (48.6)		9362 (44.0)	42 486 (47.3)	
>2	18 993 (37.6)	40 616 (22.7)		8964 (42.1)	26 397 (29.4)	
Payer						
Private/commercial insurance	26 639 (52.7)	83 643 (46.8)	<.001	9001 (42.3)	36 670 (40.8)	<.001
Medicare	16 047 (31.7)	48 182 (27.0)		8509 (40.0)	28 281 (31.5)	
Medicaid	3068 (6.1)	13 027 (7.3)		2285 (10.7)	9375 (10.4)	
Other	4809 (9.5)	33 711 (18.9)		1480 (7.0)	15 546 (17.3)	
**Hip arthroplasty**						
Total patients, No.	45 244	228 290	NA	22 235	117 072	NA
Age, mean (SD), y	65.2 (10.6)	65.9 (10.7)	<.001	66.5 (10.4)	66.4 (10.3)	<.001
Sex						
Male	18 817 (41.6)	95 832 (42.0)	.13	9350 (42.1)	50 533 (43.2)	.002
Female	26 427 (58.4)	132 458 (58.0)		12 885 (57.9)	66 539 (56.8)	
Race and ethnicity						
American Indian	56 (0.1)	352 (0.2)	<.001	31 (0.1)	134 (0.1)	<.001
Asian or Pacific Islander	343 (0.8)	2421 (1.0)		219 (1.0)	1225 (1.0)	
Black	7398 (16.4)	15 609 (6.8)		4104 (18.5)	8703 (7.4)	
Hispanic	454 (1.0)	9295 (4.1)		272 (1.2)	5224 (4.5)	
White	36 394 (80.4)	190 661 (83.5)		17 349 (78.0)	96 440 (82.4)	
Other^a^	599 (1.3)	9952 (4.4)		260 (1.2)	5346 (4.6)	
Elixhauser comorbidity index						
0	5022 (11.1)	35 809 (15.7)	<.001	2525 (11.4)	17 237 (14.7)	<.001
1-2	19 660 (43.5)	116 472 (51.0)		9956 (44.8)	57 865 (49.4)	
>2	20 562 (45.4)	76 009 (33.3)		9754 (43.9)	41 970 (35.8)	
Payer						
Private/commercial insurance	17 091 (37.8)	81 737 (35.8)	<.001	7791 (35.0)	41 654 (35.6)	<.001
Medicare	25 504 (56.4)	131 662 (57.7)		12 586 (56.6)	64 933 (55.5)	
Medicaid	1537 (3.4)	7701 (3.4)		1319 (5.9)	6225 (5.3)	
Other	1112 (2.5)	7190 (3.1)		539 (2.4)	4260 (3.6)	
**Knee arthroplasty**						
Total patients, No.	73 229	280 575	NA	33 123	144 611	NA
Age, mean (SD), y	65.2 (10.6)	66.1 (10.7)	<.001	65.7 (9.9)	66.1 (10.1)	<.001
Sex						
Male	25 946 (35.4)	100 094 (35.7)	.22	12 101 (36.5)	53 164 (36.8)	.43
Female	47 283 (64.6)	180 481 (64.3)		21 022 (63.5)	91 447 (63.2)	
Race and ethnicity						
American Indian	143 (0.2)	724 (0.3)	<.001	91 (0.3)	351 (0.2)	<.001
Asian or Pacific Islander	752 (1.0)	4835 (1.7)		620 (1.9)	3227 (2.2)	
Black	14 523 (19.8)	26 051 (9.3)		6883 (20.8)	14 184 (9.8)	
Hispanic	989 (1.4)	18 148 (6.5)		699 (2.1)	10 047 (6.9)	
White	55 712 (76.1)	216 652 (77.2)		24 300 (73.4)	108 308 (74.9)	
Other^a^	1110 (1.5)	14 165 (5.0)		530 (1.6)	8494 (5.9)	
Elixhauser comorbidity index						
0	5802 (7.9)	35 628 (12.7)	<.001	2902 (8.8)	16 686 (11.5)	<.001
1-2	33 479 (45.7)	154 571 (55.1)		15 241 (46.0)	74 806 (51.7)	
>2	33 948 (46.4)	90 376 (32.2)		14 980 (45.2)	53 119 (36.7)	
Payer						
Private/commercial insurance	30 421 (41.5)	104 655 (37.3)	<.001	12 644 (38.2)	51 609 (35.7)	<.001
Medicare	37 984 (51.9)	148 153 (52.8)		17 575 (53.1)	75 378 (52.1)	
Medicaid	2057 (2.8)	10 765 (3.8)		1779 (5.4)	8510 (5.9)	
Other	2767 (3.8)	17 002 (6.1)		1125 (3.4)	9114 (6.3)	
**Hysterectomy**						
Total patients, No.	42 053	197 058	NA	9176	53 402	NA
Age, mean (SD), y	48.9 (11.4)	51.2 (11.9)	<.001	51.0 (11.9)	51.9 (11.9)	<.001
Sex						
Male	NA	NA	NA	NA	NA	NA
Female	42 053 (100)	197 058 (100)		9176 (100)	53 402 (100)	
Race and ethnicity						
American	78 (0.2)	565 (0.3)	<.001	22 (0.1)	118 (0.2)	<.001
Asian or Pacific Islander	78 (0.2)	6919 (3.5)		300 (3.3)	2675 (5.0)	
Black	15 311 (36.4)	36 512 (18.5)		3956 (43.1)	12 042 (22.5)	
Hispanic	1325 (3.2)	24 566 (12.5)		485 (5.3)	8188 (15.3)	
White	23 409 (55.7)	115 797 (58.8)		4248 (46.3)	25 157 (47.1)	
Other^a^	1047 (2.5)	12 699 (6.4)		165 (1.8)	5222 (9.8)	
Elixhauser comorbidity index						
0	11 074 (26.3)	74 086 (37.6)	<.001	1972 (21.5)	18 291 (34.3)	<.001
1-2	20 343 (48.4)	87 829 (44.6)		4262 (46.4)	23 246 (43.5)	
>2	10 636 (25.3)	35 143 (17.8)		2942 (32.1)	11 865 (22.2)	
Payer						
Private/commercial insurance	30 563 (72.7)	124 716 (63.3)	<.001	5735 (62.5)	29 256 (54.8)	<.001
Medicare	5354 (12.7)	32 078 (16.3)		1529 (16.7)	9449 (17.7)	
Medicaid	4223 (10.0)	28 736 (14.6)		1616 (17.6)	12 245 (22.9)	
Other	1913 (4.5)	11 528 (5.9)		296 (3.2)	2452 (4.6)	
**Cesarean delivery**						
Total patients, No.	136 502	710 402	NA	55 889	279 943	NA
Age, mean (SD), y	29.9 (6.0)	30.5 (5.9)	<.001	30.6 (5.7)	31.1 (5.7)	<.001
Sex						
Male	NA	NA	NA	NA	NA	NA
Female	136 502 (100)	710 402 (100)		55 889 (100)	279 943 (100)	
Race and ethnicity						
American Indian	567 (0.4)	4050 (0.6)	<.001	315 (0.6)	1069 (0.4)	<.001
Asian or Pacific Islander	7564 (5.5)	56 016 (7.9)		3571 (6.4)	25 236 (9.0)	
Black	46 345 (34.0)	116 378 (16.4)		19 739 (35.3)	44 502 (15.9)	
Hispanic	13 676 (10.0)	131 324 (18.5)		6693 (12.0)	53 142 (19.0)	
White	62 126 (45.5)	338 036 (47.6)		23 702 (42.4)	124 158 (44.4)	
Other^a^	6224 (4.6)	64 598 (9.1)		1869 (3.3)	31 836 (11.4)	
Elixhauser comorbidity index						
0	88 624 (64.9)	554 973 (78.1)	<.001	33 550 (60.0)	200 342 (71.6)	<.001
1-2	44 581 (32.7)	148 871 (21.0)		20 783 (37.2)	75 655 (27.0)	
>2	3297 (2.4)	6558 (0.9)		1556 (2.8)	3946 (1.4)	
Payer						
Private/commercial insurance	81 349 (59.6)	421 492 (59.3)	<.001	31 239 (55.9)	152 532 (54.5)	<.001
Medicare	645 (0.5)	4376 (0.6)		351 (0.6)	1816 (0.6)	
Medicaid	50 081 (36.7)	243 210 (34.2)		21 943 (39.3)	113 304 (40.5)	
Other	4427 (3.2)	41 324 (5.8)		2356 (4.2)	12 291 (4.4)	

Abbreviations: CABG, coronary artery bypass grafting; CEA, carotid endarterectomy; NA, not applicable.

^a^ The term other in the state inpatient databases for New Jersey and Rhode Island refers to individuals of multiracial background such as White and Black, White and Asian, Black and Alaskan Native; New York State did not specify the meaning of other before 2014.

^b^ Exact values could not be reported because the Agency for Healthcare Research and Quality prohibits publication of group data if less than 10 individuals comprise any subgroup to protect participant identification and privacy.

**Figure 1.**
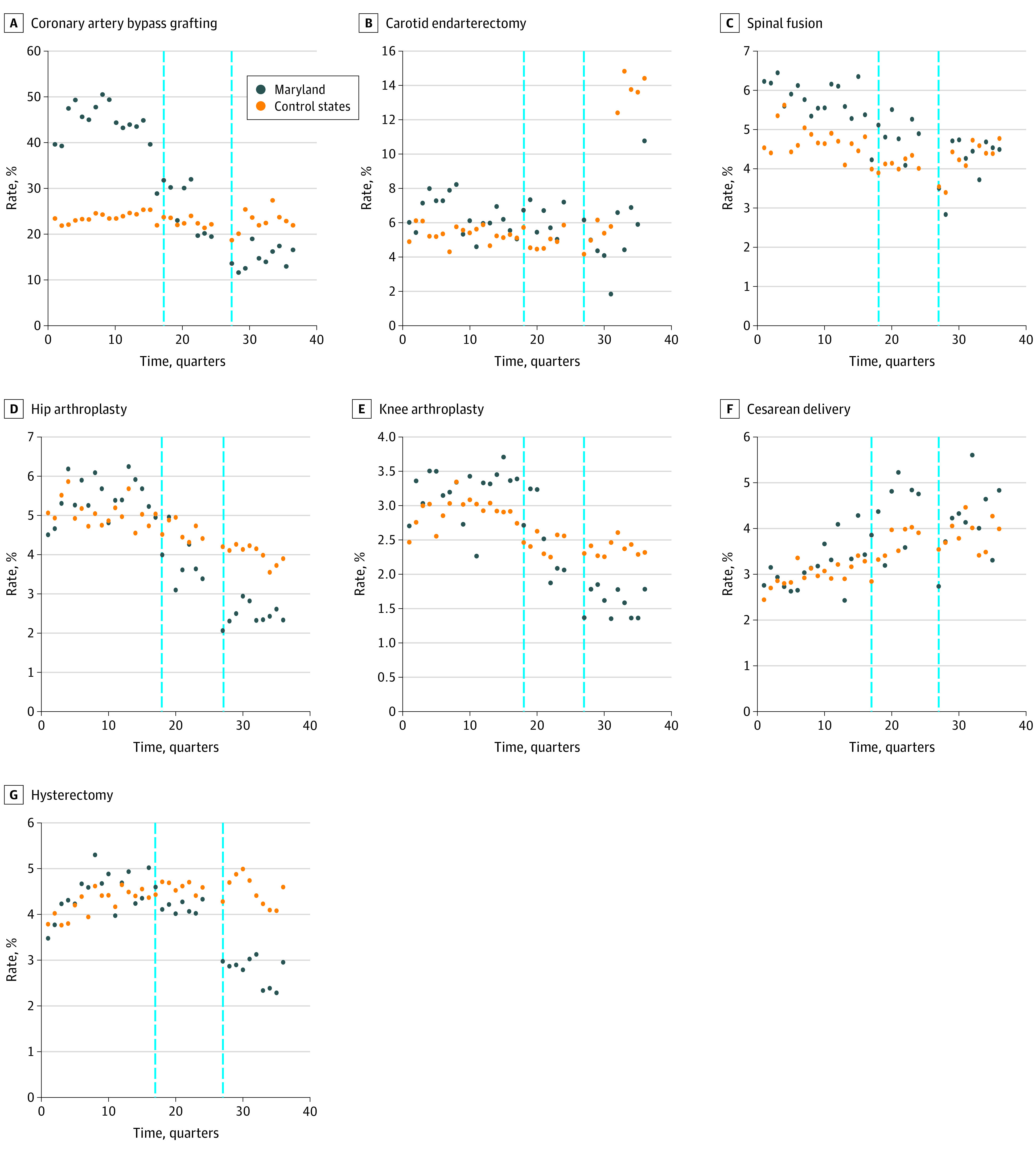
Trends in Quarterly Unadjusted Rates of Hospital-Acquired Conditions Among Patients Receiving Surgery in Maryland vs Control States, 2008-2016 Vertical dashed lines represent the implementation of the Maryland Hospital-Acquired Conditions Program (MHACP) in 2011 (left) and the Maryland all-payer model with redesign of the MHACP in 2014 (right).

**Figure 2.**
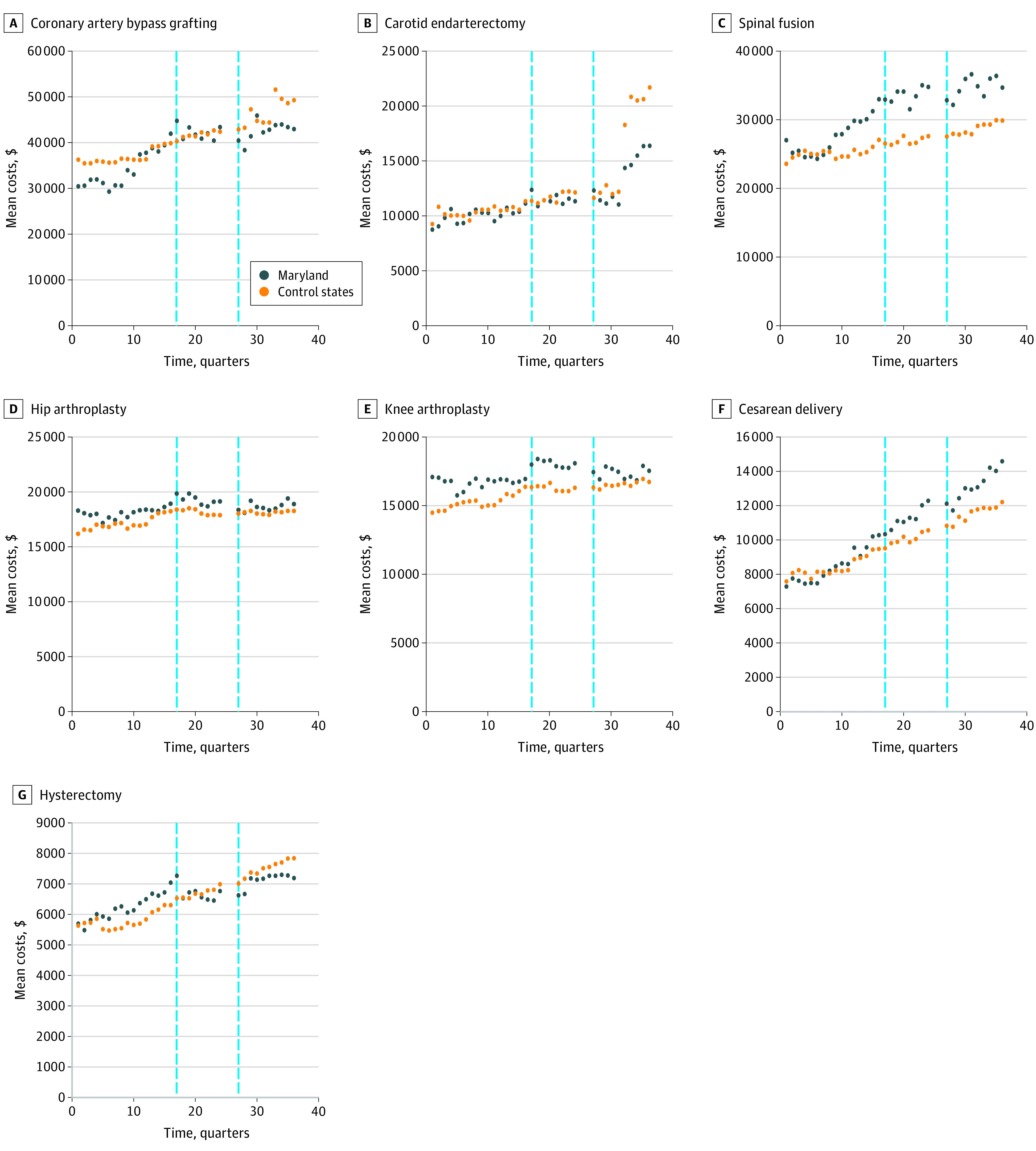
Trends in Unadjusted Mean Index Hospitalization Costs Among Patients Receiving Surgery in Maryland vs Control States, 2008-2016 Vertical dashed lines represent the implementation of the Maryland Hospital-Acquired Conditions Program (MHACP) in 2011 (left) and the Maryland all-payer model with redesign of the MHACP in 2014 (right).

Figure 1 and Figure 2 show the unadjusted quarterly rates of HACs and unadjusted quarterly means of index hospitalization costs for each surgical procedure of interest in Maryland and the control states throughout the study period. The analysis of the pre-2014 period found significant differences in baseline trends for quarterly rates of change in HACs for 3 of the 7 surgical procedures (CABG: 9.78 × 10^−3^% [95% CI, 10.95 × 10^−3^% to 8.61 × 10^−3^%; *P* < .001]; hysterectomy: 0.21 × 10^−3^% [95% CI, 0.03 × 10^−3^% to 0.39 × 10^−3^%; *P* = .03]; and cesarean delivery: −0.26 × 10^−3^% [95% CI, −0.40 × 10^−3^% to −0.12 × 10^−3^%; *P* < .001]) and in mean index hospitalization costs for 5 of the 7 procedures (CABG: $457 [95% CI, $411-$503; *P* < .001]; CEA: $25 [95% CI, $0.4-$50; *P* = .05]; spinal fusion: $312 [95% CI, $284-$341; *P* < .001]; hysterectomy: $69 [95% CI, $60-$78; *P* < .001]; and cesarean delivery: −$13 [95% CI, −$17 to −$10; *P* = .01]) (eTable 2A and eTable 2B in the Supplement). In the analysis of the pre-2011 period, no significant differences were found in baseline trends for quarterly adjusted rates of HACs for 6 of the 7 procedures, with hip arthroplasty as the exception (0.64 × 10^−3^%; 95% CI, 0.24 × 10^−3^% to 1.03 × 10^−3^%; *P* = .002) (eTable 2C in the Supplement).

Table 2 shows unadjusted differences and adjusted difference-in-differences estimates for the HAC rates and mean hospitalization costs. For HACs, the greatest changes were observed in patients who received CABG; among these patients, implementation of the all-payer model was associated with a −7.0% (95% CI, −9.4% to −4.5%; *P* < .001) decrease in the rate of HACs in Maryland compared with the control states, without adjustment for the 2011 MHACP. In the fully adjusted model, the decrease among those who received CABG was −11.3% (95% CI, −13.8% to −8.7%; *P* < .001). The rate of HACs among patients who received CEA increased less in Maryland (partially adjusted model: −2.5% [95% CI, −3.7% to −1.3%; *P* < .001]; fully adjusted model: −1.6% [95% CI, −2.9% to −0.3%; *P* = .02]). Significant but smaller decreases in HAC rates were also found among patients who received hip arthroplasty (partially adjusted model: −0.6% [95% CI, −0.9% to −0.3%; *P* < .001]; fully adjusted model: −0.8% [95% CI, −1.0% to −0.5%; *P* < .001]), knee arthroplasty (partially adjusted model: −0.6% [95% CI, −0.8 to –0.3%; *P* < .001]; fully adjusted model: −0.4% [95% CI, −0.7% to −0.1%; *P* = .01]), and cesarean delivery (partially adjusted model: −0.9% [95% CI, −1.2% to −0.6%; *P* < .001]; fully adjusted model: −1.0% [95% CI, −1.3% to −0.7%; *P* < .001]). Of note, in the partially adjusted model, differences between Maryland and the control states were significant for all surgical procedures. In the fully adjusted model, differences were significant for 5 of the 7 surgical procedures: CABG, CEA, hip arthroplasty, knee arthroplasty, and cesarean delivery.

**Table 2.** Adjusted Changes in Mean Hospitalization Costs and Rates of Hospital-Acquired Conditions in Maryland and Control States in First 3 Years After Implementation of the All-Payer Model

Surgical procedure	Difference-in-differences analysis					
	Hospitalization costs		Hospital-acquired conditions			
	Adjusted change (95% CI), $	*P* value	Partial model		Full model	
			Adjusted change (95% CI), %	*P* value	Adjusted change (95% CI), %	*P* value
CABG	−6236 (−7320 to −5151)	<.001	−7.0 (−9.4 to −4.5)	<.001	−11.3 (−13.8 to −8.7)	<.001
CEA	−730 (−1367 to −94)	.03	−2.5 (−3.7 to −1.3)	<.001	−1.6 (−2.9 to −0.3)	.02
Spinal fusion	−3253 (−3879 to −2627)	<.001	−0.5 (−0.8 to −0.1)	.02	−0.3 (−0.7 to 0.7)	.10
Hip arthroplasty	−328 (−634 to −21)	.04	−0.6 (−0.9 to −0.3)	<.001	−0.8 (−1.0 to −0.5)	<.001
Knee arthroplasty	−415 (−643 to −187)	<.001	−0.6 (−0.8 to −0.3)	<.001	−0.4 (−0.7 to −0.1)	.01
Hysterectomy	−745 (−974 to −517)	<.001	−0.5 (−0.9 to −0.1)	.02	−0.4 (−0.9 to 0.1)	.08
Cesarean delivery	−300 (−380 to −220)	<.001	−0.9 (−1.2 to −0.6)	<.001	−1.0 (−1.3 to −0.7)	<.001

Abbreviations: CABG, coronary artery bypass grafting; CEA, carotid endarterectomy.

Implementation of the all-payer model was associated with significantly smaller increases in mean index hospitalization costs in Maryland compared with the control states for all 7 surgical procedures (Table 2). Cost increases were −$6236 (95% CI, −$7320 to −$5151; *P* < .001) lower for CABG, −$730 (95% CI, −$1367 to −$94; *P* = .03) lower for CEA, −$3253 (95% CI, −$3879 to −$2627; *P* < .001) lower for spinal fusion, −$328 (95% CI, −$634 to −$21; *P* = .04) lower for hip arthroplasty, −$415 (95% CI, −$643 to −$187; *P* < .001) lower for knee arthroplasty, −$745 (95% CI, −$974 to −$517; *P* < .001) lower for hysterectomy, and −$300 (95% CI, −$380 to −$220; *P* < .001) lower for cesarean delivery.

An examination of variation in patient selection revealed differences before and after implementation of the all-payer model (eTable 3 in the Supplement). Significant shifts toward greater proportions of private/commercially insured patients were found after implementation of the all-payer model in Maryland compared with the control states for hysterectomy (1.7%; 95% CI, 0.5%-2.9%; *P* < .001) and orthopedic procedures, such as spinal fusion (4.3%; 95% CI, 3.4%-5.2%; *P* < .001), hip arthroplasty (2.5%; 95% CI, 1.7%-3.4%; *P* < .001), and knee arthroplasty (1.6%; 95% CI, 1.0%-2.3%; *P* < .001) as well as a significant shift toward more Medicaid-insured patients for cesarean delivery (3.7%; 95% CI, 3.1%-4.2%; *P* < .001). In addition, there were significant shifts away from patients with comorbidities after implementation of the all-payer model in Maryland compared with control states across all surgical procedures, ranging from −0.7% (95% CI, −0.1% to −0.5%; *P* < .001) for CABG to −3.0% (95% CI, −3.6% to −2.3%; *P* < .001) for hip arthroplasty.

## Discussion

This comparative effectiveness study found a significant association between implementation of the all-payer model in Maryland and reduced rates of cost increase across 7 common surgical procedures relative to comparison states. Implementation of the all-payer model was also associated with significantly reduced HAC rates, although there were concurrent changes in the patient mix toward a younger and healthier population. Our results are germane because inpatient hospitalization costs, estimated to be $434.2 billion in 2017, account for almost one-third of all US health care expenditures.^30^ Furthermore, in 2019, the Maryland total cost of care model was launched as an enhancement to the all-payer model. The total cost of care model includes an episode care improvement program that allows hospitals to track and link payments across care episodes, such as surgical care.

Although global budgets have been used in several countries, the US experience is limited to Maryland.^31^ The all-payer model generates a substantial efficiency incentive by uncoupling revenue from volume of services.^7,8,16^ In response, health care professionals may decrease their use of potentially avoidable services, including ambulatory-sensitive admissions and unnecessary tests and procedures.^7,8,16,17^ Periodic adjustments to hospital-specific budgets are allowed to account for any mismatch between estimated vs actual care delivery owing to unexpected shifts in patient volume.^8,32^ This provision was recently found to provide substantial protection against financial losses associated with the COVID-19 pandemic because Maryland hospitals were able to temporarily increase rates to offset losses from deferred procedures and patient volume.^33^

Previous research on the all-payer model has not identified substantial differences in acute care use or variations in primary care visits among Medicare beneficiaries after enactment.^34^ Similar null results were also reported for hospital use and spending among Medicare beneficiaries in the antecedent global budget program for rural hospitals.^27^ This finding raises questions about the extent to which the characterization of the impact of the all-payer model via Medicare claims is representative of other payers (ie, Medicaid and private/commercial). This limitation was mitigated by our analytical approach.

A plausible mechanism for the reduced rate of increase in index hospitalization costs in Maryland is likely the coexisting trend of reductions in case-mix severity. We identified statistically significant reductions in patient comorbidity burden across all surgical procedures and an increased private/commercial payer mix for orthopedic procedures (implying a younger patient mix). These findings suggest a less resource-intensive care paradigm for these surgical patients during the index hospitalization and may also explain the reductions found in HACs. Some data suggest that the use of capitated payment models may be associated with durable modifications in physician behavior, with 1 study reporting a 12% reduction in treatment intensity in the course of lower back pain treatment.^35^ Although the impact of the all-payer model may be more reliably associated with changes in health care spending rather than production costs, charges still have considerable utility as reliable proxies for health resource use, as reported in 2 studies conducted in the inpatient setting.^36,37^

Previous work has also found that implementation of the HACRP, to which control states were exposed, was not associated with any meaningful reductions in the incidence of HACs.^38^ This result likely occurred because reimbursement for the management of these HACs under the prevailing fee-for-service model was sufficient to offset the accompanying penalties. We posit that the global capitation framework of the all-payer model provides a robust economic incentive to implement and enforce the MHACP quality improvement initiatives for the following reasons: (1) provisions for additional adjustments to the hospital annual budget based on reductions in HACs (2% of hospital revenues are at risk) are included in the all-payer model; (2) the care redesign program of the all-payer model includes a hospital care improvement track, an initiative centered on improving the quality of hospital-based care; and (3) the scoring methodology and the reward and penalty structure of the MHACP relative to the HACRP include features that explicitly mandate reductions in HACs and make these reductions a priority for Maryland hospitals. As mentioned, preferential patient selection is also an associated factor.

### Limitations

This study has limitations. First, the findings are based on observational data, which limits our ability to establish causality. Although our difference-in-differences design mitigates unmeasured confounding and bias, an ideal approach would have been to use data from a matched pool of hospitals across Maryland and control states based on observable characteristics. Inability to identify useful hospital structural characteristics is a limitation of the state inpatient databases.

Second, we evaluated only the first 3 years (2014-2016) after implementation of the all-payer model, and it is possible that short-term trends differ substantially from longer-term trends in spending and avoidable complications. Third, we used *ICD-9* and *ICD-10* coding nomenclature to identify diagnoses at admission, surgical procedures of interest, and incidence of complications. Although this practice is well described in the research literature, it is possible that it introduced imprecision in our outcome estimates given the fact that our study period also encompassed the transition from *ICD-9* to *ICD-10*. However, it is unlikely that the use of *ICD-9* and *ICD-10* codes introduced a systematic bias in favor of our Maryland outcomes.

Fourth, the all-payer model included a pilot program called the Total Patient Revenue Program, which was initiated in 2010 across 10 rural hospitals in Maryland. Over our study period, 2 rural facilities had a change in ownership, and another was converted to a free-standing emergency department. Given the small mean number of beds in rural hospitals, the inability to identify changes in ownership or hospital status in the state inpatient databases, and the low likelihood of substantial surgical volumes in this care setting, we chose not to exclude facilities with a history of participation in the Total Patient Revenue Program.^39^

## Conclusions

This study of a large, diverse surgical patient population found that after the first 3 years of implementation of the all-payer model, Maryland hospitals experienced significant decreases in the probability of avoidable complications and reductions in the rate of increase in index hospitalization costs. The preferential selection of healthier patients may be associated with the lower rates of increase in hospitalization costs. Further research is needed to identify other overlapping associations or unintended consequences in care delivery (eg, changes in site of care or variation in patient experience) as the all-payer program matures. This research is particularly salient given the societal need for payment and service delivery models that are able to support the triple aim of improving care, improving population health, and reducing costs per capita in health care.

## Notes

Supplement. **eTable 1.***ICD-9* and *ICD-10* Codes Used to Identify Postoperative Complications of Interest **eTable 2.** Baseline Difference in Trends in Quarterly Rates of Hospital-Acquired Conditions and Mean Index Hospitalization Costs **eTable 3.** Adjusted Changes in Patient Characteristics in Maryland and Control States Before and After Implementation of All-Payer Model in Maryland
