# Supplementary material for: Assessment of Costs and Care Quality Associated With Major Surgical Procedures After Implementation of Maryland’s Capitated Budget Model
Source: JAMA Netw Open. 2021 Sep 24;4(9):e2126619. doi: 10.1001/jamanetworkopen.2021.26619 (PMC8463941; doi:10.1001/jamanetworkopen.2021.26619)
Supplement: Supplement. — eTable 1.ICD-9 and ICD-10 Codes Used to Identify Postoperative Complications of Interest eTable 2. Baseline Difference in Trends in Quarterly Rates of Hospital-Acquired Conditions and Mean Index Hospitalization Costs eTable 3. Adjusted Changes in Patient Characteristics in Maryland and Control States Before and After Implementation of All-Payer Model in Maryland [file jamanetwopen-e2126619-s001.pdf]

Supplementary Online Content

Aliu O, Lee AWP, Efron JE, Higgins RSD, Butler CE, Offodile AC II. Assessment of costs and care quality associated with major surgical procedures after implementation of Maryland's capitated budget model. *JAMA Netw Open*. 2021;4(9):e2126619. doi:10.1001/jamanetworkopen.2021.26619

eTable 1. *ICD-9* and *ICD-10* Codes Used to Identify Postoperative Complications of Interest

eTable 2. Baseline Difference in Trends in Quarterly Rates of Hospital-Acquired Conditions and Mean Index Hospitalization Costs

eTable 3. Adjusted Changes in Patient Characteristics in Maryland and Control States Before and After Implementation of All-Payer Model in Maryland

This supplementary material has been provided by the authors to give readers additional information about their work.

© 2021 Aliu O et al. *JAMA Network Open*.

eTable 1. *ICD-9* and *ICD-10* Codes Used to Identify Postoperative Complications of Interest

COMPLICATIONS CODES		
**Complication**	**ICD-9 CODES**	**ICD-10 CODES**
**Infectious**		
Pneumonia	480.0, 480.1, 480.2, 480.3, 480.8, 480.9, 481, 481.0, 482.0, 482.1, 482.2, 482.30, 482.31, 482.32, 482.39, 482.40, 482.41, 482.42, 482.49, 482.81, 482.82, 482.83, 482.84, 482.89, 482.9, 483.0, 483.1, 483.8, 485.0, 486.0, 487.0	J18.0, J18.1, J18.2 J18.8, J18.9, J16.0, J16.8, J15.0, J15.1, J15.20 J15.211, J15.212, J15.29, J15.3, J15.4, J15.5, J15.6, J15.7, J15.8, J15.9, J14, J13, J12.0, J12.1, J12.2, J12.3, J12.81, J12.89, J12.9, J11.00, J11.08, J10.00, J10.01, J10.08, J09.X1
Severe sepsis/septic shock	995.92, 785.52	R65.21, TX81.12XA, TX81.12XD, TX81.12XS
Post-operative infection	998.5, 998.51, 998.59	TX81.49XA, TX81.49XD, TX81.49XS, TX81.43XA, TX81.43XD, TX81.43XS, T81.42XA, T81.42XD, T81.42XS, T81.41XA, T81.41XD, T81.41XS, T81.40XA, T81.40XD, T81.40XS
**Cardiovascular**		
Cardiac arrest	427.5	I46.2 – I46.9
Acute myocardial infarction	410.00, 410.01, 410.02, 410.10, 410.11, 410.12, 410.20, 410.21, 410.22, 410.30, 410.31, 410.32, 410.40, 410.41, 410.42, 410.50, 410.51, 410.52, 410.60, 410.61, 410.62, 410.70, 410.71, 410.72,	I21.01, I21.02, I21.09, I21.11, I21.19, I21.21, I21.29, I21.3, I21.4, I21.9, I21.A1, I21.A9, I22.0, I22.1, I22.2, I22.8, I22.9

© 2021 Aliu O et al. *JAMA Network Open*.

	410.80, 410.81, 410.82, 410.90, 410.91, 410.92	
Stroke	430, 431, 433.01, 433.11, 433.21, 433.31, 433.81, 433.91, 434.01, 434.11, 434.91	I60.00, I60.01, I60.02, I60.10, I60.11, I60.12, I60.2, I60.3, I60.30, I60.31, I60.32, I60.4, I60.50, I60.51, I60.52, I60.6, I60.7, I60.8, I60.9<br>I61.0, I61.1, I61.2, I61.3, I61.4, I61.5, I61.6, I61.8, I61.9, I63.00, I63.011, I63.012, I63.013, I63.019, I63.02, I63.031, I63.032, I63.033, I63.039, I63.09, I63.10, I63.111, I63.112, I63.113, I63.119, I63.12, I63.131, I63.132, I63.133, I63.139, I63.19, I63.20, I63.211, I63.212, I63.213, I63.219, I63.22, I63.231, I63.232, I63.233, I63.239, I63.29, I63.30, I63.311, I63.312, I63.313, I63.319, I63.321, I63.322, I63.323, I63.329, I63.331, I63.332, I63.333, I63.339, I63.341, I63.342, I63.343, I63.349, I63.39, I63.40, I63.411, I63.412, I63.413, I63.419, I63.421, I63.422, I63.423, I63.429, I63.431, I63.432, I63.433, I63.439, I63.441, I63.442, I63.443, I63.449, I63.49, I63.50, I63.511, I63.512, I63.513, I63.519, I63.521, I63.522, I63.523, I63.529, I63.531, I63.532, I63.533, I63.539, I63.541, I63.542, I63.543, I63.549, I63.59, I63.6, I63.81, I63.89, I63.9
**Respiratory**		
Acute pulmonary edema	518.4	J81.0
Acute respiratory failure	518.5, 518.81, 518.82, 518.84	J95.821, J95.822, J95.89
Aspiration pneumonia	507.0	J69.0
**Renal**		

© 2021 Aliu O et al. *JAMA Network Open*.

Acute renal failure	584, 584.5, 584.6, 584.7, 584.8, 584.9	N17.0, N17.1, N17.2, N17.8, N17.9
**Coagulation**		
Deep vein thrombosis	453.4, 453.40, 453.41, 453.42	I82.401, I82.402, I82.403, I82.409, I82.411, I82.412, I82.413, I82.419, I82.421, I82.422, I82.423, I82.429, I82.431, I82.432, I82.433, I82.439, I82.441, I82.442, I82.443, I82.449, I82.491, I82.492, I82.493, I82.499, I82.4Y1, I82.4Y2, I82.4Y3, I82.4Y9, I82.4Z1, I82.4Z2, I82.4Z3, I82.4Z9
Pulmonary embolism	415.1	I26.01, I26.02, I26.09, I26.90, I26.92, I26.99
**Wound complications**		
Wound disruption	998.3, 998.30, 998.31, 998.32	TX81.30XA, TX81.30XD, TX81.30XS, TX81.31XA, TX81.31XD, TX81.31XS, TX81.32XA, TX81.32XD, TX81.32XS
**Obstetric**		
Complications of obstetric surgical wounds	674.10, 674.12, 674.14, 674.30, 674.32, 674.34, 666.00, 666.02, 666.04, 666.10, 666.12, 666.14, 666.20, 666.22, 666.24, 666.30, 666.32, 666.34	O90.0, O90.2, O72.0, O72.1, O72.2, O72.3

© 2021 Aliu O et al. *JAMA Network Open*.

eTable 2. Baseline Difference in Trends in Quarterly Rates of Hospital-Acquired Conditions and Mean Index Hospitalization Costs

A. Baseline difference in trends in quarterly adjusted rates of Hospital Acquired Conditions among patients undergoing procedures of interest in Maryland and control states. Comparisons based on pre-2014 Alternative Payment Model Intervention

	Pre-intervention difference in quarterly rate of change in Hospital-Acquired-Conditions (95% CI)	*p*
CABG	9.78×10^{-3} % (10.95×10^{-3}, 8.61×10^{-3})	< 0.01
CEA	0.08×10^{-3} % (-0.42×10^{-3}, 0.58×10^{-3})	0.75
Spine Fusion	0.13×10^{-3} % (-0.04×10^{-3}, 0.31×10^{-3})	0.14
Hip Arthroplasty	-0.09×10^{-3} % (-0.25×10^{-3}, 0.07×10^{-3})	0.28
Knee Arthroplasty	0.06×10^{-3} % (-0.07×10^{-3}, 0.20×10^{-3})	0.38
Hysterectomy	0.21×10^{-3} % (0.03×10^{-3}, 0.39×10^{-3})	0.03
Caesarean Section	-0.26×10^{-3} % (-0.40×10^{-3}, -0.12×10^{-3})	< 0.01

CABG: Coronary artery bypass grafting; CEA: Carotid endarterectomy

B. Baseline difference in trends in quarterly unadjusted mean index hospitalization costs among patients undergoing procedures of interest in Maryland and control states

	Pre-intervention difference in quarterly rate of change in mean Hospitalization Costs (95% CI)	*p*
CABG	\$457 (\$411, \$503)	< 0.01
CEA	\$25 (\$0.4, \$50)	0.05
Spine Fusion	\$312 (\$284, \$341)	< 0.01
Hip Arthroplasty	-\$1.7 (\$12, \$16)	0.81
Knee Arthroplasty	-\$3 (-\$12, \$8)	0.64
Hysterectomy	\$69 (\$60, \$78)	< 0.01
Caesarean Section	-\$13 (-\$17, -\$10)	0.01

CABG: Coronary artery bypass grafting; CEA: Carotid endarterectomy

C. Baseline difference in trends in quarterly adjusted rates of Hospital Acquired Conditions among patients undergoing procedures of interest in Maryland and control states. Comparisons based on pre-2011 Maryland Hospital Acquired Conditions Program.

	Pre-intervention difference in quarterly rate of change in Hospital-Acquired-Conditions (95% CI)	*p*
CABG	0.56×10^{-3} % (-2.0×10^{-3}, 3.12×10^{-3})	0.67
CEA	-0.14×10^{-3} % (-1.22×10^{-3}, 0.93×10^{-3})	0.79
Spine Fusion	0.14×10^{-3} % (-0.27×10^{-3}, 0.55×10^{-3})	0.51
Hip Arthroplasty	0.64×10^{-3} (0.24×10^{-3}, 1.03×10^{-3})	< 0.01
Knee Arthroplasty	-0.05×10^{-3} (-0.37×10^{-3}, 0.27×10^{-3})	0.75
Hysterectomy	0.07×10^{-3} % (-0.28×10^{-3}, 0.44×10^{-3})	0.68
Caesarean Section	-0.05×10^{-3} % (-0.37×10^{-3}, 0.27×10^{-3})	0.76

CABG: Coronary artery bypass grafting; CEA: Carotid endarterectomy

© 2021 Aliu O et al. *JAMA Network Open*.

eTable 3. Adjusted Changes in Patient Characteristics in Maryland and Control States Before and After Implementation of All-Payer Model in Maryland

A

MEDICAID		
**PROCEDURE**	**DIFFERENCE-IN-DIFFERENCES (% change)**	***p***
**CABG**	-0.9 (-1.8, -0.004)	0.05
**CEA**	0.1 (-0.8, 1.0)	0.80
**Spine Fusion**	-1.5 (-2.1, -1.0)	<0.01
**Hip Arthroplasty**	-0.6 (-1.0, -0.2)	<0.01
**Knee Arthroplasty**	-0.5 (-0.8, -0.2)	<0.01
**Hysterectomy**	0.8 (-0.1, 1.7)	0.10
**Cesarean section**	3.7 (3.1, 4.2)	<0.01

B

MEDICARE		
**PROCEDURE**	**DIFFERENCE-IN-DIFFERENCES (% change)**	***p***
**CABG**	1.1 (-0.5, 2.7)	0.19
**CEA**	0.3 (-1.5, 2.2)	0.75
**Spine Fusion**	-3.8 (-4.6, -2.9)	<0.01
**Hip Arthroplasty**	-2.4 (-3.3, -1.6)	<0.01
**Knee Arthroplasty**	-1.9 (-2.6, -1.1)	<0.01
**Hysterectomy**	-2.5 (-3.4, -1.6)	<0.01
**Cesarean section**	-0.1 (-0.2, -0.04)	<0.01

C

PRIVATE		
**PROCEDURE**	**DIFFERENCE-IN-DIFFERENCES (% change)**	***p***
**CABG**	-0.1 (-1.6, 1.4)	0.86
**CEA**	-0.7 (-2.4, 1.0)	0.43
**Spine Fusion**	4.3 (3.4, 5.2)	<0.01
**Hip Arthroplasty**	2.5 (1.7, 3.4)	<0.01
**Knee Arthroplasty**	1.6 (1.0, 2.3)	<0.01
**Hysterectomy**	1.7 (0.5, 2.9)	<0.01
**Cesarean section**	-1.2 (-1.7, -0.6)	<0.01

D

RACE (BLACK)		
**PROCEDURE**	**DIFFERENCE-IN-DIFFERENCES (% change)**	***p***
**CABG**	1.3 (0.1, 2.5)	0.03
**CEA**	1.0 (-0.3, 2.3)	0.15
**Spine Fusion**	2.7 (2.0, 3.4)	<0.01
**Hip Arthroplasty**	1.5 (0.9, 2.1)	<0.01
**Knee Arthroplasty**	0.4 (-0.1, 1.0)	0.14
**Hysterectomy**	2.7 (1.5, 3.9)	<0.01
**Cesarean section**	1.9 (1.4, 2.3)	<0.01

© 2021 Aliu O et al. *JAMA Network Open*.

E

RACE (HISPANIC)		
**PROCEDURE**	**DIFFERENCE-IN-DIFFERENCES (% change)**	***p***
**CABG**	0.03 (-0.5, 0.6)	0.93
**CEA**	0.7 (0.2, 1.3)	0.01
**Spine Fusion**	0.4 (0.1, 0.7)	0.01
**Hip Arthroplasty**	-0.2 (-0.4, 0.1)	0.13
**Knee Arthroplasty**	0.3 (0.04, 0.5)	0.02
**Hysterectomy**	-0.7 (-1.3, -0.1)	0.02
**Cesarean section**	1.5 (1.1, 1.8)	<0.01

F

COMORBIDITIES		
**PROCEDURE**	**DIFFERENCE-IN-DIFFERENCES (% change)**	***p***
**CABG**	-0.7 (-0.1, -0.5)	<0.01
**CEA**	-0.8 (-1.6, -0.1)	0.02
**Spine Fusion**	-3.0 (-3.6, -2.3)	<0.01
**Hip Arthroplasty**	-1.2 (-1.8, -0.7)	<0.01
**Knee Arthroplasty**	-2.0 (-2.4, -1.6)	<0.01
**Hysterectomy**	1.5 (0.5, 2.5)	<0.01
**Cesarean section**	-1.7 (-2.2, -1.1)	<0.01

© 2021 Aliu O et al. *JAMA Network Open*.
